# AI-Assisted Systematic Review: Humans Still Need to Review All Abstracts for Inclusion

**DOI:** 10.2196/82896

**Published:** 2026-03-19

**Authors:** Hyelin Sung, Deyana Altahsh, Scott Garrison

**Affiliations:** 1 Faculty of Medicine & Dentistry, University of Alberta, Edmonton, AB, Canada; 2 Department of Family Medicine, Faculty of Medicine & Dentistry, University of Alberta, 6-60 University Terrace, Edmonton, AB, T6G 2T4, Canada, 1 (587) 785 3012

**Keywords:** title and abstract screening, artificial intelligence, AI, large language model, LLM, machine learning, ChatGPT, GPT-5, ASReviewLab, systematic review

## Abstract

Although a general purpose (GPT-5), and a fine-tuned (ASReviewLab) artificial intelligence were able to rank abstracts for likely inclusion in a variety of Cochrane systematic reviews, some actually included studies were not highly ranked, necessitating human review of all abstracts.

## Introduction

Although systematic reviews (SRs) are the gold standard of evidence synthesis, they are time consuming, requiring evaluation of thousands of abstracts [1]. Artificial intelligence (AI) has the potential to identify and rank relevant studies for inclusion [2,3] but do they reliably rank all eligible abstracts high enough that only a subset of high-ranked abstracts require human review? We evaluated where studies actually included in published SRs fell within the “most likely for inclusion” rankings of a general-purpose AI (GPT-5), and an AI fine-tuned to identify SR-eligible studies (ASReviewLab)—assessing AI ranking reliability under worst-case constraints.

## Methods

### Overview

The most recently published 25 Cochrane SRs with ≥5 included studies were identified (as of May 5, 2025, from all subject areas), and their reported search strategies were replicated using the same databases, algorithms, and date ranges reported in each SR. We did not carry out gray literature searches, nor search databases with undefined algorithms, and limited abstracts to English. Deduplication and data export were completed using Covidence for each SR individually. Studies included in the published reviews were separated into “main results” publications and “supplementary studies” (eg, protocols, interim findings, subgroup analyses). Each publication record was separately ranked. We preidentified the AI tool as having “high-utility” if all eligible studies were placed/presented in the top 500, or top 15%, of AI-ranked abstracts (anticipated to shorten human review time by 85%).

For GPT-5 Thinking (version August 7, 2025), an AI prompt was iteratively developed to rank abstracts in order of likely inclusion. The prompt included 1) assessing whether the abstract reported on a randomized trial, 2) each SR’s PICO (Patient/Population, Intervention, Comparison, and Outcome) criteria (setting, population, intervention, comparator, outcomes), and 3) an example of an included study (ie, the main results abstract for the trial with largest enrollment). An example of the final (best performing) prompt, used for all rankings, is provided in Multimedia Appendix 1.

For ASReviewLab (version 2.1.1), due to platform differences, PICO criteria could not be provided. Rather, per this AI’s normal procedure of continually learning from human feedback, we provided it three included abstracts (those with highest enrollment), and subsequently labeled studies as included, or excluded, in the order ASReviewLab presented them. Given this interaction was time-consuming, we limited evaluation of ASReviewLab to the 10 most recent Cochrane SRs, and evaluated a maximum 500 studies for inclusion per SR.

### Ethical Considerations

All studies were identified from previously published and publicly available SRs. Therefore, no research ethics approval was required. Ethical AI use was upheld through reporting of prompts and processes for replicability, and all AI outputs were evaluated by human reviewers for accuracy.

## Results

Five SRs were excluded for having <5 included studies (ie, 25 of the 30 most recent SRs were eligible). The total number of abstracts requiring review ranged from 152 to 39,132 studies.

**Figure 1.**
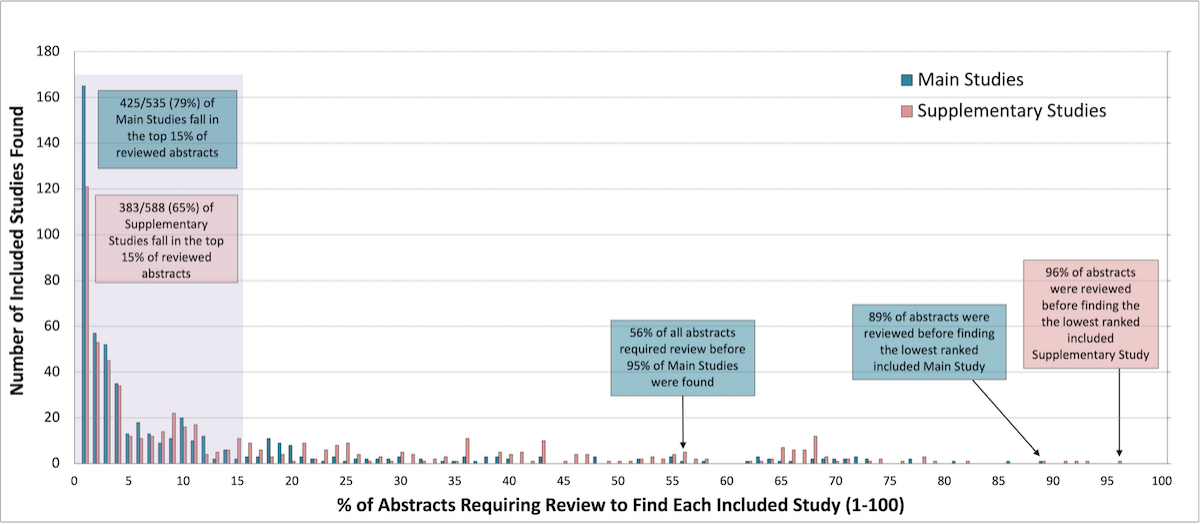
GPT-5: % of abstracts requiring review to find each included study.

For GPT-5 Thinking, 144,120 abstracts were screened, of which 1123 were included studies—comprising 535 main results publications and 588 supplementary studies. Although 79.4% (n=425/535) of main results and 65.1% (n=383/588) of supplementary studies were within the highest-ranked 15% of abstracts, 89% (n=128,266/144,120) of abstracts were more highly ranked than the lowest-ranked main results publication, and 96% (n=138,355/144,120) of abstracts were more highly ranked than the lowest-ranked supplemental study (Figure 1).

**Figure 2.**
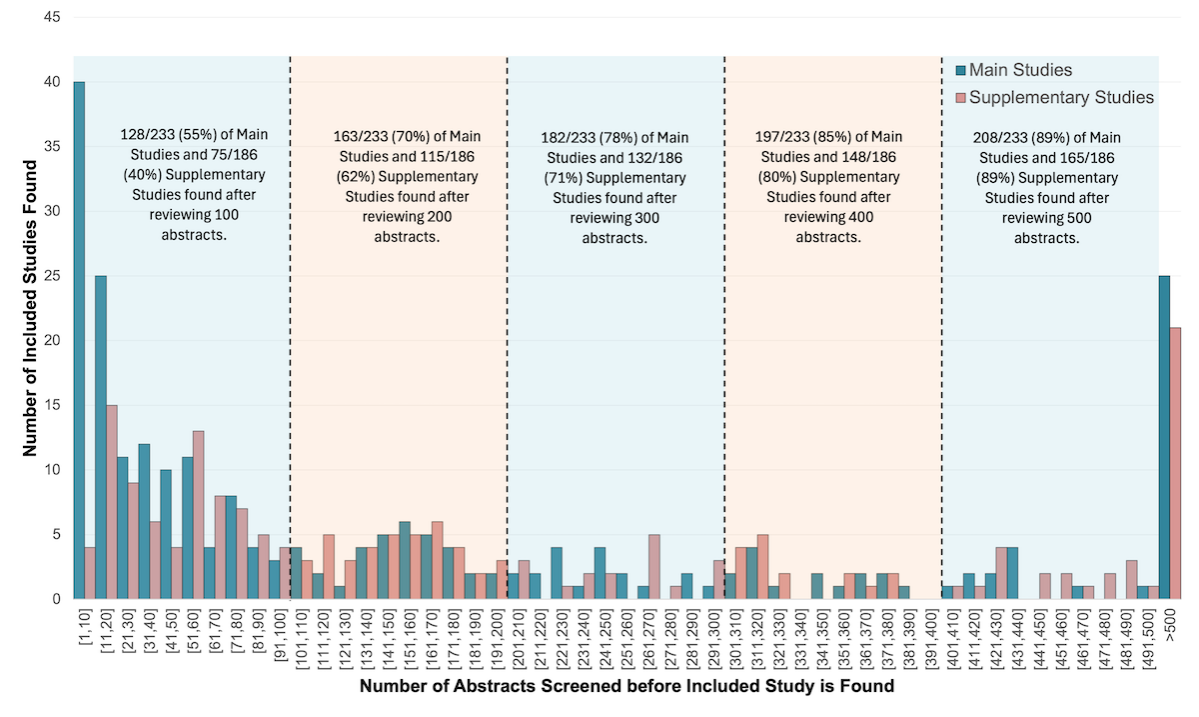
ASReviewLab: number of abstracts screened before included study is found.

For ASReviewLab, 80,298 abstracts required review, of which 419 were included studies—comprising 233 main results publications and 186 supplementary studies. Although 89% of both main results (n=208/233) and supplementary studies (n=165/186) were identified in the first 500 AI-ranked abstracts, that left 11% (n=46/419) of included studies yet to be identified (Figure 2).

## Discussion

Both AI tools ranked the majority of included studies within the first 500, or first 15% of abstracts. However, many included studies required substantially more abstracts to be reviewed before they were identified. For GPT-5, where it was possible to see where all abstracts were ranked, some included studies would have required 96% of abstracts to be reviewed before all included studies were found – making these tools unreliable, given ALL eligible studies need to be identified (ie, unacceptable tail risk).

Our findings are limited to the two AI tools utilized and to SRs with well formulated PICO questions, as is the norm for the Cochrane Collaboration. It is possible that better prompts could have been developed to improve GPT-5 performance. Had we reviewed more than 500 abstracts with ASReviewLab, we might also have found all included studies to lie within a still reasonable number of abstracts for humans to review in a timely manner – especially since reviewing more abstracts could have improved the AIs ability to recognize eligible studies. For GPT-5, there appeared to be no problem with prompt limits, domain variation, or publication types – as both main studies and supplementary studies had similar proportions of low-ranked included studies. Why some included studies received a low ranking is unclear.

Our findings align with other work suggesting AI models have potential to automate the SR process. However, the AI tools reported on here are not yet reliable enough to limit the number of abstracts human reviewers must evaluate if the goal is to find all eligible studies [4].

Our study is novel in providing a PICO-criteria-based prompt to GPT 5-Thinking, and in replicating abstract searches for multiple Cochrane reviews in order to identify all studies which should have been highly-ranked by AI tools. These Cochrane reviews additionally spanned many areas of medicine – from neonatal acupuncture to improving professional practice.

Unless performing a scoping review, where some relevant studies might be expected to be missed [5], the AI tools we examined are not yet sufficiently reliable at identifying eligible studies that lower-ranking studies do not require evaluation by a human reviewer. Doubtless this will change in time, as AI models evolve, for which further such evaluations of the ability to rank eligible studies would be worthwhile—including supplementary studies, and SRs of a non-medical nature, which pose different classification challenges.

## Supplementary material

10.2196/82896 Multimedia Appendix 1 Sample prompt to rank abstracts in order of most to least likely for inclusion.

## Abbreviations

AI: artificial intelligence
PICO: Patient/Population, Intervention, Comparison, and Outcome
SR: systematic review
