# Supplementary material for: AI-Assisted Systematic Review: Humans Still Need to Review All Abstracts for Inclusion
Source: JMIR Form Res. 2026 Mar 19;10:e82896. doi: 10.2196/82896 (PMC13002160; doi:10.2196/82896)
Supplement: Multimedia Appendix 1 [file formative-v10-e82896-s001.docx]

Sample prompt to rank abstracts in order of most to least likely for inclusion in the Cochrane Systematic Review: **Withdrawal of Antihypertensive Drugs in Older People** (*Cochrane Database Syst Rev. 2025 Mar 31;3(3):CD012572*)

Step 1:

You are acting as an expert systematic reviewer at the abstract-screening stage for a Cochrane Review.

A single Excel workbook will be supplied containing one sheet named ""abstracts"" with at least these columns:

covidence_id (unique numeric identifier)

title

abstract

(Ignore any other fields.)

Task Overview

Use the provided inclusion/exclusion criteria to evaluate each abstract against the following six fixed yes/no judgment questions. For each question, determine if the criterion is met based on the title and abstract content.

The six questions (Q1–Q6) are:

Was this a randomised controlled trial? (Based on study design criteria.)

Was the study’s setting consistent with the review’s inclusion criteria?

Was the population consistent with the review’s inclusion criteria?

Was the intervention consistent with the review’s inclusion criteria?

Was the comparator consistent with the review’s inclusion criteria?

Did the study report or collect any primary or secondary outcomes of interest to the review? (Yes if at least one outcome matches.)

For every abstract, read and interpret the full content (concatenating title and abstract), reasoning step-by-step. Do not rely on simple keyword matching—consider context, synonyms, and implications. If information is missing or unclear, use ""Can't Tell.""

Assign a 5-point confidence rating for each question, where ""Yes"" means the criterion is met (supports inclusion), and ""No"" means not met (supports exclusion):

1 = Definitely Yes – criterion clearly and explicitly met

2 = Possibly Yes – criterion likely met, but with minor ambiguity or doubt

3 = Can’t Tell – insufficient or no relevant information to judge

4 = Probably Not – criterion likely not met, based on available evidence

5 = Definitely Not – criterion clearly and explicitly not met

For Q1 specifically: Assign 'Definitely Not' (5) only if the methodology clearly states a different trial design (e.g., explicitly mentions a non-randomized design like observational or case-control). If the abstract mentions another trial design but it is unclear, or if there is no mention of study design at all, do not assign 5; instead use 3 (Can't Tell) or 4 (Probably Not) as appropriate. Do not assign 5 unless there is a clear statement of a different trial design.

Convert ratings to points: 1 → 0 (strong include), 2 → 1 (mild include), 3 → 3 (neutral/uncertain), 4 → 5 (mild exclude), 5 → 20 (strong exclude). This scoring penalizes exclusions heavily to prioritize potential includes.

Sum the six point values to produce an exclusion_score for each abstract (lower score = better, indicating higher inclusion likelihood).

Inclusion/Exclusion Criteria

Q1: Is this a randomised controlled trial (RCT), including standard RCTs with truly random allocation (e.g., computer-generated randomization, random number tables, sealed envelopes, block randomization, stratified methods, or minimization), parallel-group trials (separate arms without switching), multi-arm trials (multiple intervention groups if at least one fits comparisons like withdrawal vs continuation), cluster-randomised trials (cluster-RCTs with randomization at group levels like hospitals/clinics/wards/practices/regions/communities/facilities), or any RCT variant, reported as full texts, abstracts only, conference proceedings, unpublished data, online clinical trial results/summaries, or preprints? Consider synonyms or related terms like randomized clinical trial, randomised study, controlled trial with randomization, allocation-concealed RCT, or prospective randomized intervention study. Include regardless of sample size (small/large trials, pilot/feasibility studies), language (using translations if needed), publication status (published/unpublished, peer-reviewed or not), date/year/format (full article, abstract, letter). Exclude quasi-RCTs (quasi-randomized or pseudo-randomized trials using non-strictly random methods like alternation, date of birth, day of the week, admission sequence, medical record number, or other predictable approaches that could introduce bias), cross-over trials (crossover designs where groups switch interventions, as inapplicable to withdrawal interventions), or non-randomized designs (e.g., observational cohorts, case-control, case series, controlled before-after/CBA, interrupted time-series/ITS). If the design is ambiguous, partially described (e.g., "randomized" or "allocated" without full details), or implies possible true randomization (e.g., "assigned randomly"), err on the side of inclusion by assuming it could qualify as an RCT unless explicitly quasi-random, cross-over, or non-randomized.

Q2: Is the study's setting consistent with the review's inclusion criteria? Since there are no explicit setting exclusions, default to yes (score 1)

Q3: Is the population consistent with the review's inclusion criteria? Does the study include adults aged 50 years and over (also known as older adults/seniors/elderly/geriatric individuals/middle-aged and above, aged ≥50/50+ years, mature adults/post-middle-age participants, using cut-off of 50 to maximize inclusion of older/relevant studies especially from low/middle-income countries/LMICs), prescribed one or more antihypertensive medication(s) (also called blood pressure-lowering drugs/BP meds/anti-hypertensives/hypertension treatments, including diuretics like loop/thiazide-type/potassium-sparing/aldosterone antagonists such as furosemide/torsemide/hydrochlorothiazide/chlorthalidone/indapamide/amiloride/triamterene/spironolactone/eplerenone, beta-blockers like atenolol/carvedilol/propranolol/metoprolol, ACE inhibitors like captopril/enalapril/lisinopril/ramipril, calcium channel blockers like amlodipine/felodipine/nifedipine/verapamil/diltiazem, angiotensin II receptor antagonists/blockers/ARBs like candesartan/irbesartan/losartan/valsartan, renin inhibitors like aliskiren, alpha-blockers like doxazosin/prazosin/terazosin, or centrally-acting like clonidine/methyldopa) for hypertension (high blood pressure/elevated BP/HTN, primary/essential hypertension, or secondary if not excluded) or primary prevention of cardiovascular disease/CVD (CVD risk reduction/prophylaxis, preventing heart disease/stroke/MI/atherosclerosis/vascular events in at-risk without prior CVD), living in the community (home-based/residential/non-institutionalized/domiciliary living), residential aged care facilities (nursing homes/long-term care/assisted living/elder care residences), or hospital settings (inpatient/acute care/admitted patients)? For mixed ages, include if all ≥50, results for ≥50 subgroup presented/separable (plan to contact authors), or majority ≥50 as per mean/median age + SD/IQR (mean - SD ≥50, or 75th percentile/IQR showing ≥75% ≥50, or reported number ≥50). Include if indication is hypertension or primary CVD prevention, and <20% with baseline CVD (subclinical/prior events allowed if minority). Synonyms for population: older hypertensive adults on BP meds for primary prevention, seniors ≥50 prescribed anti-hypertensives for high BP or CVD risk reduction, geriatric participants with elevated blood pressure on diuretics/beta-blockers/ACEI/CCB/ARB/renin inhibitors/alpha-blockers/clonidine. If population description is partial, uses synonyms (e.g., "elderly hypertensives on therapy," "50+ adults for BP control/CVD prophylaxis," "aged participants with high BP meds"), or ambiguous (e.g., "hypertension patients" implying ≥50), err on inclusion unless clearly <50 majority, >20% baseline CVD, or non-hypertension/CVD prevention (e.g., secondary prevention/established CVD).

Q4: Is the intervention consistent with the review's inclusion criteria? Does the study involve withdrawal of antihypertensive medications (also known as deprescribing/discontinuation/stopping/cessation/reduction/tapering off BP-lowering drugs/meds, de-intensification of anti-hypertensive therapy/regimen, medication de-escalation for hypertension, abrupt/sudden/immediate stoppage or gradual/tapered/dose-down titration to complete withdrawal or partial dose reduction/lowering, including supervised/structured/unstructured deprescribing protocols) in older adults prescribed for hypertension or primary CVD prevention, targeting any eligible antihypertensive (diuretics like furosemide/torsemide/hydrochlorothiazide/chlorthalidone/indapamide/amiloride/triamterene/spironolactone/eplerenone acting on nephron sodium reabsorption/loop/thick ascending limb/distal tubule/collecting duct/aldosterone blockage, beta-blockers like atenolol/carvedilol blocking catecholamine receptors in heart/vasculature/bronchi/pancreas/uterus/kidney/brain/liver, ACE inhibitors like captopril/enalapril blocking renin-angiotensin system/angiotensin I to II conversion/bradykinin effects, calcium channel blockers like amlodipine/felodipine blocking L-type calcium channels/vasodilation, angiotensin II receptor antagonists/ARBs like candesartan/irbesartan blocking AT1 receptors/vasoconstriction/sodium retention/aldosterone release, renin inhibitors like aliskiren blocking angiotensinogen to angiotensin I, alpha-blockers like doxazosin/prazosin antagonizing alpha-adrenergic receptors/vasodilation/peripheral resistance reduction, clonidine centrally acting on alpha2/imidazoline receptors/sympathetic tone reduction)? Include any withdrawal method (abrupt/gradual, dose reduction to zero/partial, single/multiple meds deprescribed). Synonyms: antihypertensive deprescribing/cessation in seniors, BP med withdrawal/tapering for older hypertensives, de-intensification of anti-hypertensive regimens in ≥50 adults. Err on inclusion for any withdrawal-like intervention in eligible participants, even if partially described or using synonyms (e.g., "stopping BP drugs in elderly," "tapering anti-hypertensives for CVD risk," "med reduction for high BP"), unless explicitly non-withdrawal (e.g., dose increase/continuation without stop) or unrelated meds (e.g., non-antihypertensive).

Q5: Is the comparator consistent with the review's inclusion criteria? Does the study compare the intervention to no withdrawal of antihypertensive medications (continuation/persistence/maintenance of BP-lowering therapy/regimen, ongoing/unchanged anti-hypertensive treatment, standard/usual care without deprescribing/cessation/reduction, control arm keeping meds as is)? Include if continuation involves same antihypertensives (diuretics/beta-blockers/ACEI/CCB/ARB/renin inhibitors/alpha-blockers/clonidine). Synonyms for comparator: no deprescribing control, continued anti-hypertensive arm, maintenance therapy group. Include multi-arm trials if at least one comparison fits. Allow co-interventions (e.g., monitoring/lifestyle advice) if similar across groups. Err on inclusion unless comparator is absent, mismatched (e.g., different med switch without continuation focus), or confounds (e.g., unequal baselines/add-ons biasing groups).

Q6: Did the study collect or report any primary or secondary outcomes of interest to the review? Primary: Mortality (all-cause death/fatality/overall survival rates, cardiovascular mortality/CV death/heart-related fatality like MI/stroke/heart failure deaths). Myocardial infarction (MI/heart attack, fatal/non-fatal AMI/acute myocardial infarction, cardiac event/infarct). Adverse drug reactions and adverse drug withdrawal reactions (ADRs/side effects/harms from meds like hypotension/dizziness/electrolyte imbalance/kidney issues, withdrawal reactions/rebound effects like rebound hypertension/BP spike/withdrawal syndrome upon stopping). Secondary: Hospitalisation (all-cause admission/inpatient stay/hospital entry, cardiovascular hospitalisation/CV-related admission like for MI/stroke/heart failure, heart failure hospitalisation/HF admission/decompensation entry). Stroke (cerebrovascular accident/CVA, fatal/non-fatal ischaemic/hemorrhagic stroke, transient ischaemic attack/TIA/mini-stroke). Blood pressure/BP (systolic/SBP/top number, diastolic/DBP/bottom number before/after withdrawal, mean arterial pressure/MAP/average BP). Falls (tumbling/slipping/accidental drops, fall incidents/events in older adults). Quality of life/QoL of participants/carers/families (well-being/HRQoL/life satisfaction via validated instruments like EQ-5D/EuroQol-5D/SF-36/Short Form-36, including physical/mental/emotional/social domains). Success rate of withdrawal from antihypertensive drugs over short-term (≤12 months/1 year or less, ability to complete study off meds without restarting). Success rate of withdrawal over long-term (>12 months/more than 1 year, sustained deprescribing without resumption/resistance to restarting pre-withdrawal treatment). No restriction on follow-up duration. Include if any outcome is mentioned, even indirectly or partially (synonyms: death rates, MI incidences, drug harms/withdrawal effects, admissions, strokes/TIAs, BP changes, fall risks, QoL scores, deprescribing success/sustained off-meds). Since outcomes do not determine eligibility, err heavily on inclusion—if any relevant outcome is implied, planned, measured but unreported (plan to check protocols/contact authors), or vaguely described (e.g., "deaths monitored," "BP assessed post-stop," "falls tracked," "QoL evaluated"), answer yes; only no if explicitly none match or focus solely on unrelated measures (e.g., cost without clinical outcomes).

Before Starting: Extract required data from uploaded excel file without truncating any abstracts or titles

For Each Abstract: Internally reason step-by-step for ratings, citing specific evidence from the title/abstract.

Output File: Use Python with pandas to read the input file, add new columns for Q1–Q6 (as integers 1-5), and exclusion_score (as integer). Do not modify existing columns or textual content. Sort rows by exclusion_score, then by covidence_id ascending. Write to a new Excel file named ""ranked_screening.xlsx"" with a single sheet ""screened.""

Code Guidelines: Import pandas as pd. Use pd.read_excel() for input, perform calculations in a DataFrame, and to_excel() for output. Handle common errors (e.g., NaN values in abstracts by treating as ""Can't Tell""). If the dataset is large (>500 rows), process in batches if needed.

Delivery: After generation, provide a direct download link to the output file or describe how to access it (e.g., ""The file ranked_screening.xlsx is now available for download."").

Summary: After output, add a brief summary note (e.g., ""Processed X abstracts. Score range: min=Y, max=Z, avg=W."") unless an irrecoverable error occurs (then report only the error).

No Additional Output: Present no other commentary. Begin processing only when the dataset is attached and criteria are specified.

Step 2:

Now, let’s carefully work through the following example: Covidence ID: 924

Effect of discontinuation of antihypertensive treatment in elderly people on cognitive functioning-the DANTE Study Leiden: A randomized clinical trial

Importance: Observational studies indicate that lower blood pressure (BP) increases risk for cognitive decline in elderly individuals. Older persons are at risk for impaired cerebral autoregulation; lowering their BPmay compromise cerebral blood flowand cognitive function. Objective(s): To assess whether discontinuation of antihypertensive treatment in older persons with mild cognitive deficits improves cognitive, psychological, and general daily functioning. Design, Setting, Andparticipants: Acommunity-based randomized clinical trial with a blinded outcome assessment at the 16-week follow-upwas performed at 128 general practices in the Netherlands. A total of 385 participants 75 years or older with mild cognitive deficits (Mini-Mental State Examination score, 21-27) without serious cardiovascular diseasewhoreceived antihypertensive treatmentwere enrolled in the Discontinuation of Antihypertensive Treatment in Elderly People (DANTE) Study Leiden from June 26, 2011, through August 23, 2013 (follow-up, December 16, 2013). Intention-to-treat analyseswere performed from January 20through April 11, 2014. Intervention(s): Discontinuation (n = 199) vs continuation (n = 186) of antihypertensive treatment (allocation ratio, 1:1). Main Outcomes and Measures: Change in the overall cognition compound score. Secondary outcomes included changes in scores on cognitive domains, the Geriatric Depression Scale-15, Apathy Scale, Groningen Activity Restriction Scale (functional status), and Cantril Ladder (quality of life). RESULTS Compared with 176 participants undergoing analysis in the control (continuation) group, 180 in the intervention (discontinuation) group had a greater increase (95%CI) in systolic BP (difference, 7.36 [3.02 to 11.69]mmHg; P = .001) and diastolic BP (difference, 2.63 [0.34 to 4.93]mmHg; P = .03). The intervention group did not differ from the control group in change (95%CI) in overall cognition compound score (0.01 [-0.14 to 0.16] vs -0.01 [-0.16 to 0.14]; difference, 0.02 [-0.19 to 0.23]; P = .84). The intervention and control groups did not differ significantly in secondary outcomes, including differences (95%CIs) in change in compound scores of the 3 cognitive domains (executive function, -0.07 [-0.29 to 0.15; P = .52], memory, 0.08 [-0.12 to 0.29; P = .43], and psychomotor speed, -0.85 [-1.72 to 0.02; P = .06]), symptoms of apathy (0.17 [-0.65 to 0.99; P = .68]) and depression (0.14 [-0.20 to 0.48; P = .41]), functional status (-0.72 [-1.52 to 0.09; P = .08]), and quality-of-life score (-0.09 [-0.34 to 0.16; P = .46]). Adverse events were equally distributed. Conclusions and Relevance: In older persons with mild cognitive deficits, discontinuation of antihypertensive treatment did not improve cognitive, psychological, or general daily functioning at the 16-week follow-up.Copyright © 2015 American Medical Association. All rights reserved.
